# Ecosystem Services in Agricultural Landscapes: A Spatially Explicit Approach to Support Sustainable Soil Management

**DOI:** 10.1155/2014/483298

**Published:** 2014-01-30

**Authors:** Mohsen Forouzangohar, Neville D. Crossman, Richard J. MacEwan, D. Dugal Wallace, Lauren T. Bennett

**Affiliations:** ^1^Department of Forest and Ecosystem Science, The University of Melbourne, 4 Water Street, Creswick, VIC 3363, Australia; ^2^Commonwealth Scientific and Industrial Research Organisation (CSIRO) Ecosystem Sciences, PMB 2, Urrbrae, SA 5064, Australia; ^3^Future Farming Systems Research Division, Department of Environment & Primary Industries, P.O. Box 3100, Bendigo Delivery Centre, VIC 3554, Australia; ^4^Agriculture Productivity Group, Department of Environment & Primary Industries, 32 Lincoln Square, North Carlton, VIC 3053, Australia

## Abstract

Soil degradation has been associated with a lack of adequate consideration of soil ecosystem services. We demonstrate a broadly applicable method for mapping changes in the supply of two priority soil ecosystem services to support decisions about sustainable land-use configurations. We used a landscape-scale study area of 302 km^2^ in northern Victoria, south-eastern Australia, which has been cleared for intensive agriculture. Indicators representing priority soil services (soil carbon sequestration and soil water storage) were quantified and mapped under both a current and a future 25-year land-use scenario (the latter including a greater diversity of land uses and increased perennial crops and irrigation). We combined diverse methods, including soil analysis using mid-infrared spectroscopy, soil biophysical modelling, and geostatistical interpolation. Our analysis suggests that the future land-use scenario would increase the landscape-level supply of both services over 25 years. Soil organic carbon content and water storage to 30 cm depth were predicted to increase by about 11% and 22%, respectively. Our service maps revealed the locations of hotspots, as well as potential trade-offs in service supply under new land-use configurations. The study highlights the need to consider diverse land uses in sustainable management of soil services in changing agricultural landscapes.

## 1. Introduction

That soils are fundamental to a wide range of ecosystem services needs to be acknowledged to avoid further soil degradation and to identify sustainable land-use change. Several recent conceptual works have used an ecosystem service approach to highlight the importance of soils to the sustained prosperity and welfare of humankind [1–4]. Nonetheless, decreases in the supply of soil ecosystem services like water quality regulation and soil structure stabilization are symptomatic both of ongoing loss of soil natural capital and of ongoing disregard of the full range of soil services in production systems [3, 4].

Better integration of ecosystem service knowledge in soil management systems requires holistic yet straightforward approaches to the quantification and mapping of soil ecosystem service supply [5]. Rutgers et al. [6] and van Wijnen et al. [7] are recent examples, although these studies did not provide approaches for quantifying changes in multiple soil ecosystem services in response to changing management—a critical knowledge gap recognised by Haines-Young et al. [8].

Full consideration of all soil ecosystem services at landscape to regional scales will rarely be possible, necessitating firstly the identification of priority services and secondly the nomination of soil properties to represent these services. Bennett et al. [9] identified 11 “final” soil ecosystem services that are directly utilised to benefit humans (versus supporting “intermediate” services). Priority services are identified as those that offer the greatest benefit within that landscape context but that also act as likely “surrogates” for the provision of other services [9]. All soil services are essentially aggregates of soil processes, where processes are inputs, losses, transfers, and transformations of material and energy [2, 10]. For example, the intermediate soil service “organic matter cycling” (nomenclature of [9]) is the result of various processes (litter comminution, decomposition, and humification), which are expressed as rates. Full temporal quantification of multiple process rates at landscape scales is not feasible. Instead, soil properties (e.g., soil organic carbon content) can be used to represent the end-point of processes/services (e.g., carbon sequestration) and changes in soil properties used as an indication of change in potential service supply [11].

Accurate prediction of changes in soil services/properties will rely both on the direct measurement of properties and on the use of soil-specific biophysical models. In this context, changes in soil properties are simulated continuously in response to changes in soil processes as a result of interactions among soil, water, vegetation, and the atmosphere [12]. There are many soil simulation models, some of which have been recommended for soil services assessment [13]. For example, Aitkenhead et al. [14] introduced the MOSES model for quantitative assessment of selected ecosystem services calibrated for a range of soils in the UK. In Australia, Agricultural Production System Simulator (APSIM) was developed as a modelling framework, consisting of soil, plant, and management component modules, to simulate diverse production systems specific to Australian soils and conditions [15, 16]. APSIM has considerable potential as a tool for modelling changes in soil properties [13] because soil properties are simulated continuously in response to changes in weather, management, and vegetation [15, 16]. It has been applied to the prediction of changes in soil organic carbon (SOC) content as a result of changes in land management [17–19]. In addition, APSIM has been successfully tested in the simulation of soil water balance [20–22].

Soil simulation models are traditionally point-based, whereas soil ecosystem services are best represented as continuous surfaces for decision-making. Interpolation is required and kriging offers an objective and rigorous approach to interpolating between spatially-distributed data points and, thus, to mapping soil services [23, 24]. The use of kriging [25] to produce soil property maps has been demonstrated frequently for SOC [26–29]. Although many studies applied these techniques at field scales with high sample densities [26, 28], some dealt with sparser sampling schemes at much broader scales. For example, Kumar and Lal [27] estimated the spatial distribution of SOC across an area of 117599 km^2^ using 920 sample points (~ one sample per 128 km^2^). Similarly, Zhang et al. [29] produced a map of SOC for the Republic of Ireland based on kriging interpolations using an average sample density of two per 100 km^2^. Clearly, in the context of broad-scale changes in soil properties, a key requirement is to choose a sampling intensity that accommodates cost and logistical constraints, but that is also sufficient for capturing change trends at appropriate scales within accepted levels of error. Here, while demonstrating the advantages of kriging from grid samples for regional estimations of soil properties, McBratney and Webster [24] concluded that the best sampling strategy would be a regular grid scheme with the highest affordable number of observations.

We used a novel combination of methods including field sampling, biophysical modelling (APSIM), and spatial interpolations (e.g., kriging) to produce landscape-scale maps indicating changes in the supply of soil services from current to future management scenarios. We selected a study area in northern Victoria, south-eastern Australia, to illustrate the utility of this approach for considering priority soil services in decisions relating to changing configurations of land-use practices at landscape scales. We focused on two priority services, namely, carbon sequestration and water storage. We used a landholder defined scenario of future land-use configurations (involving increased complexity/diversity) to predict effects of land-use change on the spatial distribution of two soil properties (hereafter “indicators”), namely, SOC content (indicating carbon sequestration) and volume of water stored in soil (indicating water storage). Our aims were to quantify and map the distribution of service indicators (representing priority services) under both current and future land-use configurations and to demonstrate the utility of our approach in supporting land-use decisions by highlighting locations where management resources could be targeted to maintain or enhance the supply of priority soil services [11, 30–32].

## 2. Methods

### 2.1. Approach Overview

**Figure 1:**
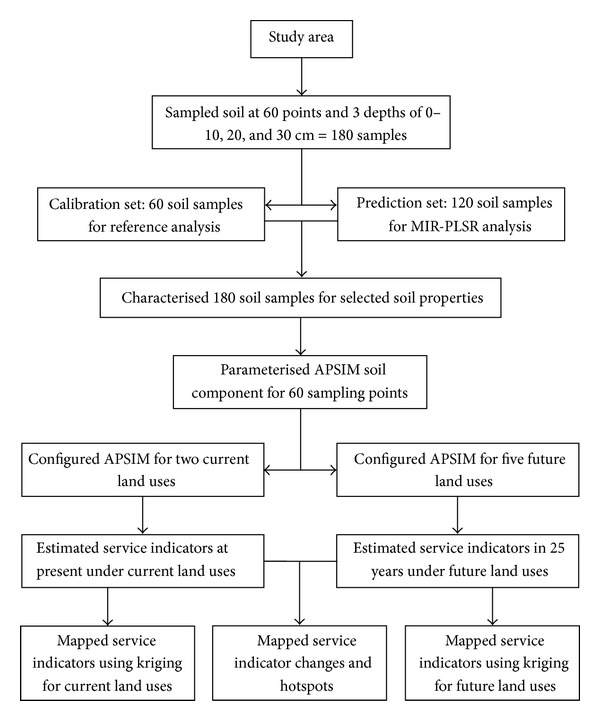
Approach overview, summarising the multiple steps leading to the production of indicator maps (representing priority services) under current and future land-use configurations.

Our unique approach (Figure 1) included representative sampling of soils across the study area according to a geostatistically-valid design, analysis of selected soil properties in a subset of reference soil samples, development of mid-infrared calibration models for the prediction of soil properties in the remaining samples, parameterising and configuring the APSIM model to predict indicators (representing priority services) under current and future land-use scenarios, and applying spatial interpolations (kriging) in a GIS environment to produce maps of current and of likely future changes in the distribution of these indicators across the entire study area. In addition, a hotspot map of soil services change was produced to help evaluate the likely trade-offs and opportunities arising from future land-use configurations.

### 2.2. Study Area

**Figure 2:**
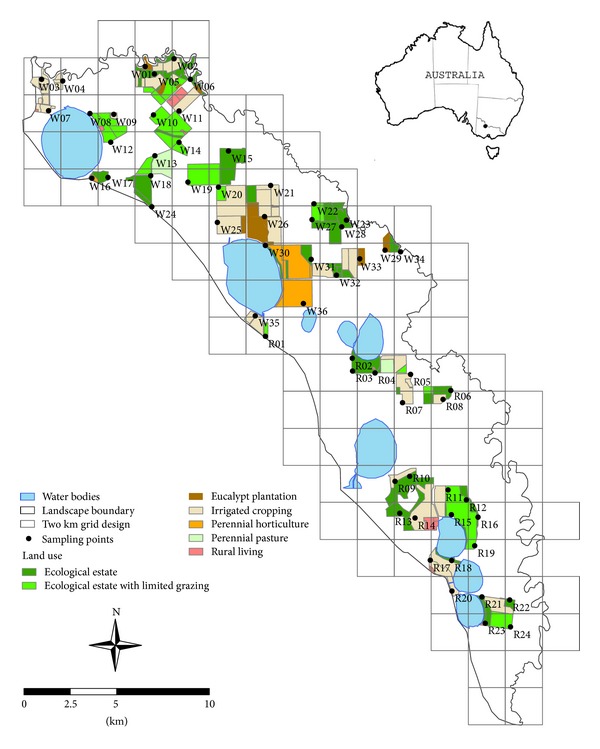
Study area location, the distribution of proposed land uses in the future (25-year) land-use scenario (covering a total area of 6,441 ha) and the associated irregular-grid used to sample soils in this study.

This study encompasses a case-study landscape of 30,200 ha (302 km^2^) in northern Victoria, between 35° 24.53′ and 35° 41.23′ S and 143° 37.72′ and 143° 54.22′ E, south-eastern Australia (Figure 2). The region has a warm grassland climate (Australian Bureau of Meteorology—http://www.bom.gov.au/iwk/climate_zones/) and typically experiences hot and dry summers and cold winters and an average annual rainfall of 370 mm falling mostly in winter and spring (June to November). Mean monthly minimum temperatures range from 3.3°C (August) to 18.4°C (January), and mean monthly maximum temperatures range from 13.1°C (July) to 35.8°C (January; climate data based on 25-year data from nearby Swan Hill and Kerang weather stations).

There is no detailed soil survey of the study area. According to the Australian Soil Classification [33] and the Victorian GMU250 geomorphology database [34], the dominant soil orders are Sodosols (or Solonetz in World Reference Base (WRB) soil classification) and Vertosols (or Vertisols in WRB soil classification), generally in the western and eastern sides of the study area, respectively. The region contains several water bodies, some of which are recognised as part of the Ramsar Convention on Wetlands, [35]. The Victorian Land Use Information System 2009 dataset [36] lists the principal land uses within the study area as domestic livestock grazing, mixed farming and grazing, and cereal dry cropping. The landscape has been extensively cleared for agriculture, and the remaining native vegetation is highly fragmented and/or restricted to riparian zones. Historical clearing and intensive agriculture in this landscape have contributed to degradation of soil capital through erosion, compaction, and localised salinisation [37, 38].

Many land parcels within the study landscape have come under the ownership and management of a single entity and are part of a 25-year plan to extensively reconfigure land uses for improved agricultural production and for improved environmental stewardship. Associated changes in the location and types of agricultural and restoration practices will directly and indirectly affect the supply of soil services [31], making this an ideal landscape for examining service changes under likely future scenarios. The 25-year plan is specific to 6,441 ha, a subset of the 30,200 ha study area, and includes proposed land uses on a continuum from less intensively managed systems (“ecological estate” with and without minor grazing and eucalypt plantation) to more intensively managed systems (mainly irrigated cropping, limited perennial pasture, and perennial horticulture; Figure 2).

### 2.3. Soil Sampling and Analysis

We used a 2 km grid as the starting point for the soil sampling design on the basis that grid sampling is often recommended when ordinary kriging is to be used for spatial distribution predictions [28]. Such a sampling scheme minimises the kriging variance, that is, the standard error of prediction [39, 40]. A 2 km sampling grid was proposed in this study because of its capacity to provide an adequate sampling intensity within cost and logistical constraints. When overlain on the study landscape, the 2 km grid yielded c. 60 strata (segments) that coincided with accessible land parcels covered by the abovementioned 25-year plan. One sampling point was chosen at the centre of each of these strata or from the nearest available location, leading to an irregular-grid sampling scheme consisting of 60 sampling points (Figure 2).

Each sampling point was located in the field, and an area of 10 by 10 m was defined for sampling. Within each sampling-point area, three randomly located subsamples were collected from each of three soil depths (0–10, 10–20, and 20–30 cm) using a soil auger of 8.25 cm internal diameter (AMS Inc., ID, USA). Subsamples from each depth were bulked to give a single composite sample per depth per sample-point area. Separate intact cores (custom-made 7.5 × 10 cm core) were also collected for bulk density determination.

The 180 soil samples (60 sampling points × 3 depths) were air-dried and passed through a 2 mm sieve in preparation for mid-infrared (MIR) spectral analysis. A subset of 60 soil samples were analysed for selected chemical and physical properties (see Table 1), and MIR spectroscopy was then used to estimate these properties in the remaining 120 samples. For MIR spectral scanning, a sub-sample (approximately 7 g) of each soil was finely ground to an approximate particle size of less than 0.1 mm diameter using a vibrating mixer ball mill (Retsch, Haan, Germany) set on a vibrational frequency of 20 s^−1^ for 3 min.

### 2.4. Analysis of Soils Using MIR Spectroscopy and Partial Least-Squares (PLS) Regression

We used a Spectrum-One (PerkinElmer, Wellesley, MA, USA) Fourier transform MIR spectrometer to collect diffuse reflectance MIR spectra of the complete set of 180 soils following a methodology described elsewhere [45, 46]. Soil MIR spectroscopy provides the possibility of rapid, inexpensive, and simultaneous characterisation of various chemical properties in large numbers of samples [2, 45, 47]. Moreover, the repeatability and reproducibility of this technique among different laboratories have been found to be superior to the performance of conventional soil analyses [2]. prior to partial least-squares (PLS) regression, principal component analysis (PCA) was performed to detect any spectral outliers and to select a calibration subset of 60 soil samples for PLS model development. PCA revealed one sample as a spectral outlier, which along with the calibration subset was included in the reference soil property characterisation (Table 1).

The calibration models for predicting SOC, pH, electrical conductivity (EC), and percent sand, silt, and clay were developed between MIR spectra (predictor variables) and reference soil property values (response variables) based on the method introduced by Haaland and Thomas [48], which is popular in quantitative soil MIR analysis [47]. The softwares GRAMS/AI, GRAMS IQ, and IQ Predict, which are part of the GRAMS spectroscopy software suite (Thermo Fisher Scientific, Waltham, MA), were used for PCA and PLS-regression model building and predictions. The MIR-PLS prediction models from the calibration subset were applied to the remaining 119 samples (see Forouzangohar et al. [45, 46]), and these predicted soil properties were used in the remainder of the study.

### 2.5. APSIM for Predicting Indicators to Represent Priority Services

Two priority soil ecosystem services were identified for consideration, namely, carbon sequestration and water storage. These two services were nominated by the landholder as central to their land-use decision making (e.g., potential involvement in both carbon and water markets) and were identified as priority services for the benefit of humans and the environment by Aitkenhead et al. [14]. We chose to estimate current and future capacity to supply these services using two indicators, namely, SOC content and soil water storage. In this case study, the service “water storage” can include water added as irrigation. As such, this service represents capacity of the soil to retain a scarce resource (water), either from natural or artificial sources, in the landscape, and to reliably provide that resource for plant growth.

Key APSIM modules used in this study were SOILWAT [20, 49] for simulating soil water dynamics and SOILN [15, 19] for soil organic carbon. In addition, the MANAGER components were utilised to control land-use configurations (as below), and the plant growth modules (Agpasture, Soybean, Oat, Wheat, Lucerne, and *Eucalyptus grandis*) were used to simulate the production systems corresponding to current and future land uses.

Two scenarios of indicator predictions were examined for the study landscape: one based on the current distribution of land uses and another based on a proposed future land-use configuration over the next 25 years (Figure 2). To isolate the effects of land-use change, climate change over the 25-year period was assumed to be negligible although some models predict lower rainfall and higher temperatures [50] in the study area, which could affect our indicator predictions. Data from a recent 25-year period (1985 to 2009) were used to represent climate in 25-years-time. In addition, to further avoid effects of interannual climate variability, daily weather data were defined for a “typical year” comprised of 12 “typical” months, each based on the average monthly rainfall and the distribution of rain days from two nearby weather stations (Swan Hill and Kerang, located, resp., to the north and south of the study area). Daily weather data for these weather stations were sourced from the SILO climate database (http://www.longpaddock.qld.gov.au/silo/) hosted by the Queensland Climate Change Centre of Excellence [51].

To represent the current land-use scenario (time = 0 years), APSIM was configured to simulate two land uses:

1. dry cropping system, as a continuous winter cereal cropping simulated by the Wheat module, with removal of soil surface plant residues 30–45 days after harvest in preparation for opportunistic sowing in late autumn (leaving the soil surface bare for 4-5 months),
2. pasture/grassland under intensive grazing, simulated by the Agpasture module, with regular fortnightly grazing to a remaining herbage of 500 kg/ha.

In addition, to represent the future land-use scenario (time = 25 years), APSIM was configured to simulate five new land uses (Figure 2):

1. ecological estate (protected for restoration of perennial native vegetation, predominantly grasslands and shrublands), simulated by the Agpasture module, with no plant biomass removal,
2. ecological estate with limited grazing, simulated by the Agpasture module, with two grazing events per year to a remaining herbage of 1000 kg/ha,
3. irrigated cropping system, as summer-winter rotational cropping systems, simulated by the Soybean, Oats, and Wheat modules of crops, assuming no-tillage systems, and including no removal of soil surface plant residues after harvest,
4. irrigated permanent lucerne stand (3 year ley), simulated by the Lucerne module, assuming regular harvest at flowering and removal of 95% of harvested plant material,
5. eucalypt plantation, simulated by the Egrandis and Agpasture modules using the Canopy module to account for light and water competition between trees and intertree herbage (including no removal of plant biomass); note that APSIM currently lacks modules for tree species other than eucalypts, so, we treated limited areas of perennial horticulture as eucalypt plantations for the purposes of this study (i.e., perennial horticulture is not considered further).

Five soil types of the Mallee region in north-western Victoria, Australia, were chosen from the APSIM soil database to underpin APSIM modelling. The soils were chosen from those generic sites that had similar characteristics to sampled soils and were further parameterised for SOC, EC, pH, particle size distribution, and bulk density using our measures of the 60 reference soils.

### 2.6. Mapping Service Indicators

Ordinary kriging was used to estimate spatial distribution of service indicators over the study area under both current and future land-use scenarios. We used ArcGIS 10.0 (ESRI Inc., CA, USA) to produce distribution maps of indicators (SOC content and soil water storage) based on kriging between the 60 sample points. Here, the average distance between neighbouring sample points was 1300 m, so that a lag size of 1300 m was used in the semivariogram models (which depict the spatial autocorrelation of the sample points and define the weights of the kriging functions). With 10 lags of this size, semivariograms of up to 13,000 m were fitted for each service indicator under each scenario. The exponential functions were best fitted through semivariogram modelling, and nuggets were set to zero (i.e., it was assumed that there was no error for model-derived values at each sample point). Using the trend analysis tool, second-order polynomial trends were identified and fitted to remove directional trends in SOC content data. As required, log transformations were used to better approximate normal distributions of input data. Given a 2 km sampling grid, a range of 2,300 m was identified as appropriate when fitting the semivariogram models in all cases. In spatial interpolation of SOC content, each kriging average was obtained using a four-sector searching neighbourhood, searching for a maximum of 5 and a minimum of 2 points in each sector. In mapping soil water storage any points beyond the 2300 m range was not involved in predictions.

### 2.7. Mapping Hotspots of Service Change

Changes in the two service indicators between current and future scenarios were normalized to a 0-1 scale through linear scaling [52]. This allowed comparison of relative estimated changes in the two priority services, with a combined value of −2 indicating maximum negative change in both and of +2 indicating maximum positive change in both. These combined values were then used to produce a “hotspot” map (where a hotspot indicates positive change in both [30]) encompassing the 60 sampling points.

### 2.8. Assessing Uncertainties of Spatial Interpolations

The overall reliability of the indicator prediction surfaces (maps) over the entire study area was assessed using the mean prediction error and the standardized Root-Mean-Square Error (standardized RMSE). A mean prediction error close to zero (i.e., relative to the magnitude of the average prediction error) and a standardized RMSE close to one indicate relatively unbiased predictions of service supply/change. The reliability of the kriging model predictions for specific points over the study area was expressed as the ratio of performance deviation (RPD), which is the ratio of natural variation in the sample set (standard deviation, SD) to the size of the average prediction error (i.e., the higher the RPD, the more reliable the prediction [53]). An RPD value of <1.4 indicates poor performance, RPD > 1.4 indicates acceptable model performance for qualitative to semiquantitative analysis, and RPD > 2.0 indicates excellent performance [54].

## 3. Results and Discussion

### 3.1. Soil Properties

There was significant variation in measured soil properties across the study area (Table 1). For example, SOC concentration to 10 cm depth ranged from 0.5 to 4.5% over distances of c. 10 km in the northern sections. Generally, SOC concentrations were greatest in northern and eastern sections (>2%) and least in central sections (<1%). The pH values ranged from slightly acidic (5.2 at 30 cm depth) to alkaline (9.3 at 30 cm depth), and EC ranged from 0.04 to 10.7 dS/m, reflecting patterns of localised surface salinization. Similarly, soil texture varied across the study area, with percent clay in the top 30 cm ranging from 6 to 61% and sand from 16 to 93% (Table 1).

### 3.2. Service Indicators under Alternative Land Uses

Simulations over a twenty-five-year period for a predominant clay soil in the study area predicted strong potential for increases in service indicators under the five new land uses. For example, mean SOC content of 30 t/ha under current land uses was predicted to increase under four of the new land uses, the highest being an estimated 38 t/ha under irrigated no-till cropping (Table 2). Increases in mean soil water storage were only predicted where new land uses involved irrigation (Table 2). However, increases in the ratio of aboveground biomass to soil water storage were predicted from 0.8 and 0.9 kg/m^3^ under current land uses to 2.2 kg/m^3^ under new irrigated land uses and to 2.3, 3.9, and 84.5 kg/m^3^ for nonirrigated new land uses of ecological estate with grazing, ecological estate without grazing, and eucalypt plantation, respectively (Table 2).

### 3.3. Soil Organic Carbon Content: Current Status and Future Projections

On average, soil to 30 cm depth at the 60 sampling points under current management stored 35 t/ha soil organic carbon (“SOC”; range 13–102 t/ha; Table 3). The greatest current SOC stores (>55 t/ha) were at the northern end of the study landscape (sampling points W1, W2, W3, W4, and W7; see Figure 2 for point locations) where sampling points were adjacent to remnant native eucalypt woodlands along the Little Murray River. Isolated pockets of low relative carbon stores (<20 t/ha) were scattered throughout the study area and were associated with general cropping, mixed farming, and grazing (W8, W10, W12, W18, W36, R10).

**Figure 3:**
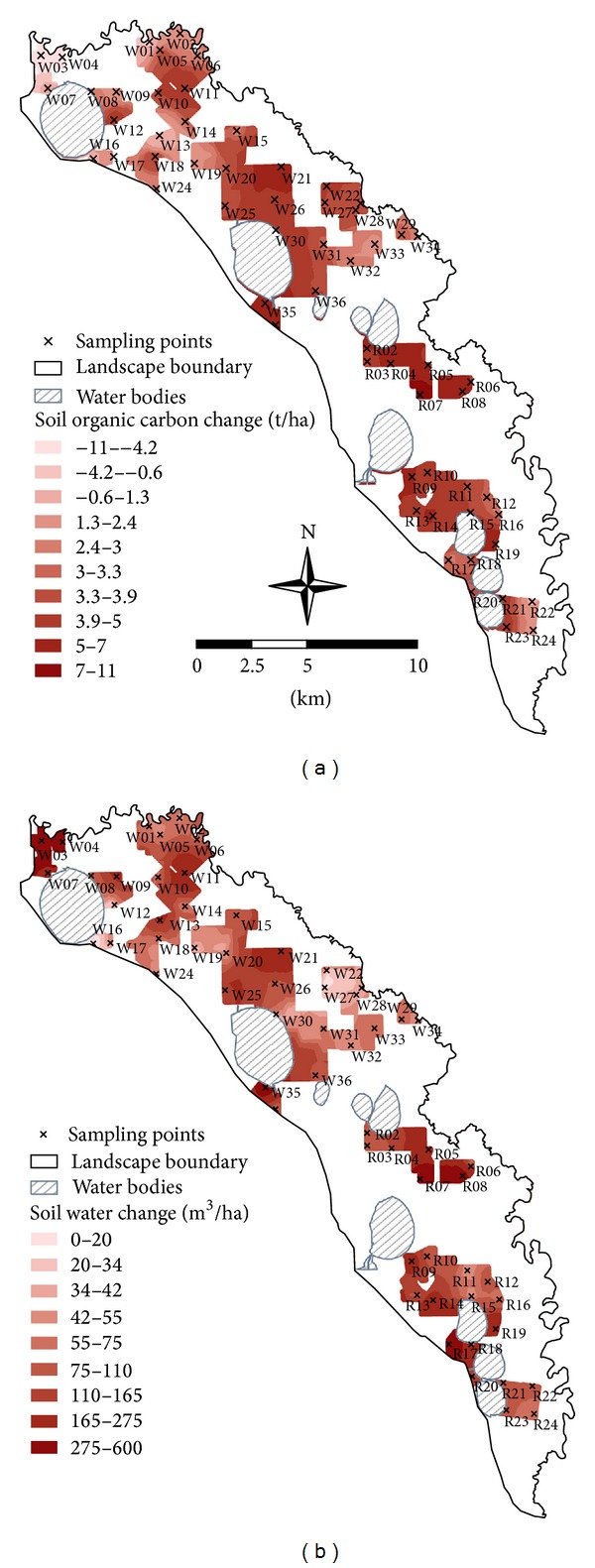
Predicted changes in service indicators, (a) soil organic carbon content and (b) soil water storage, to 30 cm depth in the study area associated with changes from current (time = 0) to future (time = 25 years) land-use scenarios. Each prediction surface represents differences in the supply of services between an average day under the current scenario and an average day under the future scenario. (a) Mean prediction error: 0.04; standardized RMSE: 1.06; average prediction error: 2.4; RPD: 1.5; (b) mean prediction error: −4.8; standardized RMSE: 1.09; average prediction error: 127; RPD: 1.2.

Predictions of soil carbon change under the future land-use scenario indicated a mean increase over the 25-year period of 3 t/ha to 30 cm depth at the 60 sampling points (Table 3). Over the 6,441 ha covered by the 25-year plan, this translated to an increase of 17,938 t SOC (i.e., change from 165,119 to 183,057 t; Table 3). Simulations indicated a range of potential changes in SOC of up to ±11 t/ha (Figure 3(a), Table 3).

Spatial predictions of SOC changes were acceptably reliable (RPD > 1.4; Figure 3(a)) and highlighted potential risks and opportunities in soil carbon management [25, 30]. For example, decreases of up to 11 t C/ha were predicted near the northern end (Figure 3(a)), corresponding with the greatest current SOC stores (>55 t/ha). Reasons for the high relative current SOC here were unclear, although they could relate to the close proximity to remnant perennial vegetation and/or to time since clearing (unknown). Nonetheless, this study's predictions indicate that the planned change in land use from dry cropping to zero-tillage irrigated cropping systems will not prevent SOC decreases, and that alternative land uses should be examined to conserve this carbon content if sustaining SOC content is a priority. Elsewhere, predicted decreases of c. 4 t C/ha near sampling point W13 in the northern section (Figure 3(a)) were associated with a planned land-use change from regular grazing to an irrigated lucerne system, involving regular removal of plant material from the soil surface. Given the already low carbon stores at this sampling point (28.4 t/ha), alternative land uses that retain rather than remove plant material might be considered.

Landscape locations that show high potential increases in SOC might also be considered for targeted management. For example, locations of maximum predicted increases (e.g., R7, W21, and W35; Figure 3(a)) were associated with changes from intensive agriculture to a zero-tillage irrigated cropping system with no plant residue removal. These data thus support strong recommendations for adoption of no-till farming by Lal et al. [55] as an option for enhancing SOC storage in cropland soils of relatively low current SOC content (<30 t C/ha). Another example of an opportunity to increase SOC stores was indicated at sampling point W12. Here, the soil was a loamy sand of very low SOC content (13.2 t/ha), and a land-use change from mixed farming with intensive grazing to ecological estate with limited grazing led to predicted increases of 5.7 t C/ha (43%) over 25 years (Figure 3(a)).

Overall, changes from intensive agriculture to ecological estate, ecological estate with limited grazing, and eucalypt plantations were associated with moderate increases in SOC of 1.3 to 5 t C/ha over the 25-year period (Figure 3(a)). This is consistent with previous findings of measured SOC increases with conversion from traditional croplands to perennial grasslands (e.g., [56–58]). Kätterer et al. [56] reported an average of 0.4 t/ha/yr increase in SOC content over 30 years when a cropland was converted back to grassland in a Swedish farm, although their study involved a wetter (542 mm/yr) and cooler (mean annual temperature of 5.1°C) climate and double the starting SOC content. In this study's landscape, limited rainfall and very hot and dry summers are likely to be limiting factors for grassland growth, as simulated by the APSIM-Agpasture module. Nonetheless, in terms of percent gain in SOC through conversion of degraded lands to natural grasslands, our results show agreement with previously reported limits of 15–20% increases [56, 57, 59].

### 3.4. Soil Water Storage: Current Status and Future Projections

Current water storage of soils reflected the predominance of clay-textured soils in the 6,441 ha covered by the 25-year plan. Total water stored to 30 cm soil depth on an average day under the current scenario was estimated at 2,745,342 m^3^ (Table 3). On average, soils stored 587 m^3^/ha water without irrigation. This value ranged from 179 m^3^/ha for a loamy sand soil under regularly-grazed pasture (sample point W12) to 843 m^3^/ha in a clay soil under dryland cereal cropping (sample point R7).

Land-use changes in the 25-year plan were predicted to increase water storage by 22% (i.e., to a mean water storage of 719 m^3^/ha and a total storage capacity of 3,362,834 m^3^; Table 3). Similarly, modelled changes in average daily soil water storage over the entire study landscape ranged from 0 to 463 m^3^/ha but were mostly >110 m^3^/ha (Figure 3(b)). Modelled soil water storage values under current and future land-use scenarios were consistent with APSIM-generated published works [21, 22]. For instance, volumetric soil water in the top 30 cm of selected soil profiles was predicted and observed to be between 0.1 and 0.4 water volume/total volume (c. 300 to 1200 m^3^/ha) in the nearby Murray-Darling Basin [22].

Predicted increases in soil water storage were associated with changes to irrigated no-till systems, and, to a lesser degree, increases in perenniality. Irrigated no-till cropping systems add water from outside the system and can increase water retention through a mulching effect associated with retained plant residues. Mean predicted increases in mean water storage in soils under this land-use change were 54%, from 669 m^3^/ha to 1,029 m^3^/ha (i.e., deepest red areas for points R5, R7-R9, R14, R17, R19, W3, W4, W7, W9, W11, W21, and W35 in Figure 3(b)). Increases in soil water storage over the 25-year period were also indicated for land uses that increased perenniality, namely, eucalypt plantation and ecological estate (with or without grazing). In these land uses, increases of 8–15% in average daily water storage were apparently due to processes affecting water infiltration and retention and were perhaps tempered by increased losses through evapotranspiration associated with the establishment of woody species [60]. This is also consistent with studies that have indicated that the reverse land-use change (i.e., from woody native vegetation to cropping) was associated with decreased soil water-holding capacity [61].

### 3.5. Relationships among Soil Ecosystem Services

**Figure 4:**
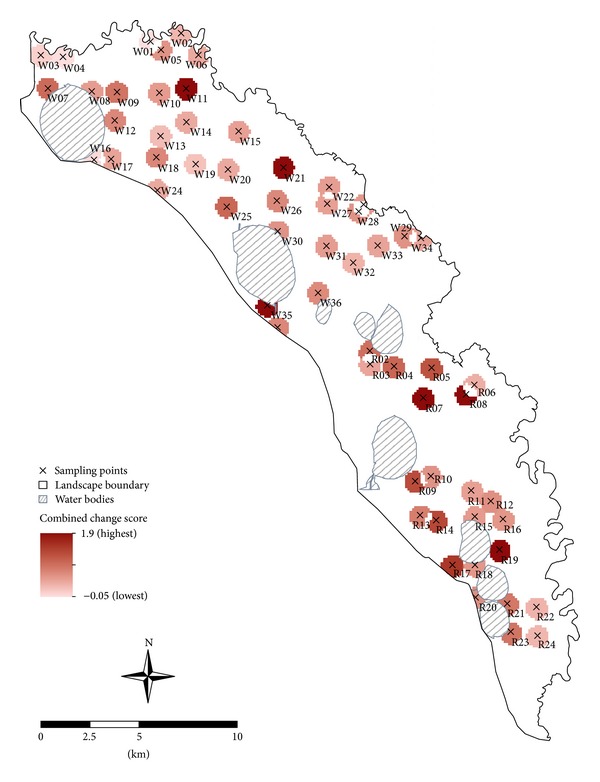
“Hotspot” map indicating combined relative change in the two service indicators (soil organic carbon content and soil water storage) from current (time = 0) to future (time = 25 years) land-use scenarios at each of 60 sampling points. Potential combined scores range from −2 (maximum negative change in both indicators) to +2 (maximum positive change in both indicators).

The indicator change maps highlight locations both of potential complementarities (synergies) among priority services, as indicated by mutual increases with changed management, and of potential trade-offs among services, as indicated by marked increases in one service but decreases in another. Overall, we found mostly positive (or “synergic”; [62]) relationships among our priority soil services (Figure 4). This suggests that they increased at the same time, due either to simultaneous responses to the same land-use change or to positive interactions [62]. In this context, hotspots are located at points where the supply of multiple soil services can be mutually and markedly improved [30].

This study found several potential hotspots as indicated by high combined service change scores (>1 to 2; Figure 4). For example, land-use change to irrigated no-till cropping led to substantial estimated increases in both soil water storage and SOC content at sampling points R7, R8, R9, R14, R17, R19, W11, W21, and W35 (Figure 4). However, the study also highlighted at least six instances of possible trade-offs between services as indicated by very low combined change scores (<0.2). Four of these cases were located at sampling points W1, W3, W4, and W13, where future predictions flagged likely reductions in SOC stocks. However, we doubt this is due to any causal relationships among the services, but, as discussed in Section 3.3, this appears to be due to the relatively high initial SOC contents at these points (except W13), which were not sustained by the new land uses. Similarly, the low combined change score at W13 was mainly due to predicted decreases in SOC associated with regular removal of plant material under the planned irrigated lucerne system. Elsewhere, the low combined change score at W16 could be attributed to negligible change in land-use practices and thus in service provision under unchanged climatic conditions.

### 3.6. Approach Limitations

We acknowledge limitations in our approach. Our spatial interpolation method of choice, kriging from point data, carries some limitations. Prediction statistics were acceptable for SOC (mean prediction error 0.04, standardised RMSE 1.06, and RPD 1.5) but were less convincing for soil water storage (mean prediction error −4.8, standardised RMSE 1.09, and RPD 1.2). In particular, the low RPD for soil water storage indicated nonreliable estimations for at least some locations perhaps due to greater spatial variation in soil water distributions than SOC.

In addition, the APSIM model could not equally represent our diversity of land-use changes. For example, APSIM plant modules have not been specifically developed for diverse native plant systems (i.e., our “ecological estate”) and for perennial horticulture systems. Moreover, it was not feasible to fully parameterise the APSIM soil components for the 60 sampling points in this study. Instead, we relied on the APSIM soil database to provide default values for some of our modelling components. This leads to uncertainties, the quantification of which is beyond the scope of this study. Future work will examine the implications of various assumptions, used not only in the APSIM modelling but also in the entire approach, through detailed sensitivity analyses.

## 4. Conclusion

We demonstrated a broadly applicable approach for quantifying and mapping service indicators (representing priority soil ecosystem services) in an agricultural landscape in south-eastern Australia. A feasible sampling intensity was combined with soil MIR spectral analysis, biophysical modeling, and spatial interpolations to provide estimates of two soil indicators under both current and future land-use scenarios. We showed that under the future land-use plan, the supply of the two priority soil services (based on indicators) would likely increase. The modelled maps provide a basis for supporting decisions about alternative land uses by indicating “hotspot” locations where there are mutual increases in the supply of soil ecosystem services or locations where there is a risk to the supply of some services. Such knowledge informs the management of multiple services at a range of scales from paddock to landscape and region, both in response to markets for particular services and to requirements or incentives for providing multiple benefits (see, e.g., the proposed “cobenefits index” in Australia's recent “Carbon Credits Bill”; Commonwealth of Australia, 2011).

The proposed approach could be useful in designing strategies for supporting sustainable soil management in this landscape. Coordinated management of multiple land parcels within a landscape, rather than isolated management of single parcels, offers potential to maintain supply of priority services at the landscape scale by avoiding risks and harnessing opportunities through matching soils with particular land-use practices. Introducing new land uses in any given landscape not only offers scope for increasing landscape complexity [63, 64] but also offers more flexibility in decisions relating to landscape-level delivery of multiple services. This type of flexibility and responsiveness will be needed as additional factors like climate change further confound the challenge to managing soils sustainably in changing agricultural landscapes.

## Figures and Tables

**Table 1:** Analysed soil chemical and physical properties of 60 sampling points (*n* = 60) at each of three depths from the study area in northern Victoria, south-eastern Australia. Data were produced through conventional analysis methods as well as mid-infrared spectroscopy*.

Soil properties	Min	Max	Mean	SD	Median
Organic carbon (%)^a^					
0–10 cm	0.3	4.5	1.3	0.8	1.2
10–20 cm	0.2	2.4	0.8	0.4	0.7
20–30 cm	0.1	1.6	0.6	0.3	0.5
pH_CaCl_2__^b^					
0–10 cm	5.4	8.7	7.1	0.7	7.1
10–20 cm	5.6	9.3	7.5	0.7	7.6
20–30 cm	5.2	9.3	7.7	0.9	7.8
EC (dS/m)^c^					
0–10 cm	0.04	5.46	0.68	1.01	0.27
10–20 cm	0.05	9.80	1.09	1.62	0.49
20–30 cm	0.04	10.72	1.60	2.08	0.75
Clay (%)^d^					
0–10 cm	6	60	40	10	42
10–20 cm	11	61	43	10	46
20–30 cm	6	61	46	12	49
Sand (%)^d^					
0–10 cm	25	93	48	13	45
10–20 cm	17	84	43	14	38
20–30 cm	16	93	39	17	35
Bulk density (g/cm^3^)					
0–10 cm	1.0	1.6	1.3	0.1	1.3
10–20 cm	1.2	1.6	1.4	0.1	1.4
20–30 cm	1.2	1.6	1.4	0.1	1.4

^a^Dry combustion and Walkley-Black methods [41].

^b^pH in 0.01 M CaCl_2_, 1 : 5 extraction ratio [42].

^c^EC in 1 : 5 water extraction ratio [43].

^d^Hydrometer method [44].

*Conventional analysis methods were applied to 61 reference samples (i.e., 61 out of the 180 study samples), and MIR spectroscopy was used to predict properties in the remaining samples.

**Table 2:** Predicted service indicators under both current and new land uses for a predominant clay soil in the study landscape. Values were derived from 25-year simulations based on average properties of the clay soil to 30 cm depth.

	Soil organic carbon (t/ha)	Soil water storage (m^3^/ha)	Aboveground biomass to soil water storage kg/m^3^ ^a^
Current land uses			
Dry cropping	30^b^	760	0.8
Intensive grazing	30^b^	680	0.9
Future land uses			
Ecological estate	34	710	3.9
Ecological estate with grazing	33	690	2.3
Eucalypt plantation	34	750	84.5
Irrigated no-till cropping	38	1200^c^	2.2
Irrigated permanent lucerne	27	1170^c^	1.1

^a^Aboveground biomass data is derived from APSIM simulations.

^b^Initial soil organic carbon content in equilibrium (modelling assumption).

^c^Soil water topped up by irrigation.

**Table 3:** Estimates of service indicator at 60 sampling points (0–30 cm depth) and over the entire study area, under current and future (25-year) land-use scenarios. Overall changes in each indicator were based on differences between supply on an average day under the current scenario and on an average day in 25-year-time under the future scenario.

Indicator	Sampling points				Entire study area^a^
	Min	Max	Mean	SD	
Soil organic carbon (t/ha)					
Current	13	102	35	17	165,119 t
Future	18	92	39	14	183,057 t
Change	−11	11	3	4	17,938 t
Soil water storage (m^3^/ha)					
Current	179	843	587	156	2,745,342 m^3^
Future	189	1220	719	251	3,362,834 m^3^
Change	0	463	132	146	617,492 m^3^

^a^Study area of 6,441 ha.
